# Outcome after decompressive craniectomy in older adults after traumatic brain injury

**DOI:** 10.3389/fmed.2024.1422040

**Published:** 2024-07-08

**Authors:** Thomas Kapapa, Stefanie Jesuthasan, Franziska Schiller, Frederike Schiller, Dieter Woischneck, Stefanie Gräve, Eberhard Barth, Benjamin Mayer, Marcel Oehmichen, Andrej Pala

**Affiliations:** ^1^Neurosurgical Department, University Hospital Ulm, Ulm, Germany; ^2^Neurosurgical Department, Hospital Landshut, Landshut, Germany; ^3^Section Interdisciplinary Intensive Care Medicine, University Hospital Ulm, Ulm, Germany; ^4^Institute for Epidemiology and Medical Biometry, University of Ulm, Ulm, Germany; ^5^Neurosurgical Department, Military Hospital Ulm, Ulm, Germany

**Keywords:** brain edema, demographic change, risk, survival rate, elderly

## Abstract

**Objective:**

Globally, many societies are experiencing an increase in the number of older adults (>65 years). However, there has been a widening gap between the chronological and biological age of older adults which trend to a more active and social participating part of the society. Concurrently, the incidence of traumatic brain injury (TBI) is increasing globally. The aim of this study was to investigate the outcome after TBI and decompressive craniectomy (DC) in older adults compared with younger patients.

**Methods:**

A retrospective, multi-centre, descriptive, observational study was conducted, including severe TBI patients who were treated with DC between 2005 and 2022. Outcome after discharge and 12 months was evaluated according to the Glasgow Outcome Scale (Sliding dichotomy based on three prognostic bands). Significance was established as *p* ≤ 0.05.

**Results:**

A total of 223 patients were included. The majority (*N* = 158, 70.9%) survived TBI and DC at discharge. However, unfavourable outcome was predominant at discharge (88%) and after 12 months (67%). There was a difference in favour of younger patients (≤65 years) between the age groups at discharge (*p* = 0.006) and at 12 months (*p* < 0.001). A subgroup analysis of the older patients (66 to ≤74 vs. ≥75 years) did not reveal any significant differences. After 12 months, 64% of the older patients had a fatal outcome. Only 10% of those >65 years old had a good or very good outcome. 25% were depending on support in everyday activities. After 12 months, the age (OR 0.937, *p* = 0.007, CI 95%: 0.894–0.981; univariate) and performed cranioplasty (univariate and multivariate results) were influential factors for the dichotomized GOS. For unfavourable outcome after 12 months, the thresholds were calculated for age = 55.5 years (*p* < 0.001), time between trauma and surgery = 8.25 h (*p* = 0.671) and Glasgow Coma Scale (GCS) = 4 (*p* = 0.429).

**Conclusion:**

Even under the current modern conditions of neuro-critical care, with significant advances in intensive care and rehabilitation medicine, the majority of patients >65 years of age following severe TBI and DC died or were dependent and usually required extensive support. This aspect should also be taken into account during decision making and counselling (inter-, intradisciplinary or with relatives) for a very mobile and active older section of society, together with the patient’s will.

## Introduction

The United Nations foresee a significant change in the demographic composition of our societies. According to this, almost 10 billion people will be living on earth by 2050 (1). In this composition, significantly more people aged ≥65 years will live on earth than children of ≤5 years of age (1). Furthermore, the older adults of this century differ considerably from their peers of the last century or those at the turn of the millennium. Today’s older adults are less likely to be retired, are more often still members of the working population, and are more likely to engage in active leisure activities such as physical exercise or outdoor activities, which has led to an increase in general activity over time (2). Today’s older adults attach great importance to their own autonomy. This means that they want to live a self-determined life, and express and realise their own independent wishes and will (3). In order to maintain part of this autonomy, more older adults are traffic participants than in the past and use cars or bicycles to maintain their mobility (4, 5). Compared to the past, there is therefore a considerable difference between the chronological and psycho-social (biological) age of older adults, with a significant trend towards more participation and activity. On the one hand, the demographic change with increasing number of older adults is associated with an increase in hospital admissions and older adults requiring treatment of age-related illnesses (6–11). This includes traumatic brain injury (TBI) which has a strong relationship to age (12, 13). On the other hand, demographic change with an increasing proportion of an active and self-determined older population makes it necessary to reflect on the limits of surgical indications according to biological more than the chronologic age (1, 14–18). Demographic change has an impact on therapies and decision-making in hospitals (19–21).

Importantly, patients older than 65 years have higher rates of mortality and morbidity after trauma (12, 13, 22, 23). In particular, the occurrence of previous illnesses plays a significant role in the prognosis of older adults after TBI. The concept of frailty plays an important role here, although frailty and chronological age should not be used synonymously (24, 25). People with pre-existing cardiac diseases and anticoagulation have a worse prognosis than younger people or older people without pre-existing medical conditions and pre-medication (26). The question therefore arises as to whether known treatment algorithms for TBI with particular emphasis on chronological age (24, 27–33) should be reviewed with regard to demographic change with more active and self-determined biological age (25). Especially in light of the fact that there are no specific instructions or guidelines for older adults with severe TBI (24, 34–36).

TBI itself is a condition that affects people all over the world and is challenging for the public health as well as for the socio-economic aspects (37). According to estimates, the incidence of TBI for all age groups is 349 (CI 95%: 96.2–1,266) per 100,000 person-years (38). There is evidence of an increasing incidence of visits to the emergency department and in-patient hospital visits in the group of elderly post-TBI patients (12, 37, 39, 40). The mean age of TBI patients has increased steadily over the past 50 years (31, 41). In the United States, the incidence of TBI with in-hospital treatment was found to be highest among those >75 years of age (41). Given the above mentioned conditions of an aging society with a growing proportion of older adults who are at risk of TBI and high mortality, the question arises of how best to use the finite resources of a health care system and of the society (13, 42, 43). In relation to severe TBI and its surgical and intensive care treatment, ethical, medical and social aspects play a serious role in decision-making. The problem with an age-based policy is that, although the risk is associated with chronological age, the individual (biological) risk of an adverse outcome is not known and is not solely dependent on chronological age (44, 45).

Entering more specific into TBI treatment, Decompressive craniectomy (DC) is able to decrease refractory increased intracranial pressure (ICP) after TBI and ensure survival (46–50). However, studies about DC in older adults are rare (12, 51–53). This study is focussed on the outcome after DC in patients aged ≤65 years versus patients aged >65 years of age. These results are intended to enrich the knowledge, to increase scientific data in the field and evidence for current and future decision-making in older adults who potentially need DC. This data should be used to inform patients and relatives in the difficult conflict between the opportunities for the biological and risks of chronological age as well as for expectations and perceptions of biological age.

## Patients and methods

### Study design

This investigation took the form of a retrospective, multi-centre, descriptive, observational study that included patients who underwent DC between January 2005 and December 2022. The patients were treated in three different hospitals with three independent neurosurgical departments. All neurosurgical departments work at a tertiary level of care with access to a specialised neurocritical care unit. Differences in the experience of neurosurgical treatment were excluded by the authors. The treatment of patients with TBI was based on the applicable national and international guidelines in all departments. During the period under review, the population in the catchment area totalled a mean of 1,431,726 (SD: 32340.9) inhabitants (54). The study was approved by the local ethics committee of the University of Ulm, Germany (No. 439/17 and No. 63/23). Due to the retrospective nature of this study, the ethics committee granted a waiver of consent.

### Patients and treatment procedures

All patients included were initially treated at the three study hospitals or sent as a secondary transport from a different hospital. Level of consciousness before surgery was rated on the Glasgow Coma Scale (GCS) (55). Pattern of cerebral injuries in patients with TBI were specifically rated by the Marshall scoring system (56). Treatment procedures are described earlier and do not differ between earlier and the actual cohort (57). Briefly summarized, the basis for treatment with DC was a severe TBI. Severe TBI was defined clinically by a GCS value of <10 (58–61) and/or by imaging with the Marshall Score of >2 (62). DC was performed as primary [after evacuating an intracranial mass lesion, e.g., an acute subdural haemorrhage (63)] or secondary [in order to control raised ICP in diffuse TBI (36, 64)]. Escalating conservative therapy measures according to the recent TBI guidelines were started with an ICP > 20 mmHg (35, 57, 65). An ICP value >25 mmHg over 15 min without reduction by positioning measures, deepening of sedation, hyperventilation or hyperosmolar solutions such as mannitol or 10% NaCl solution was defined as a refractory ICP elevation. Patients of all ages were included in this retrospective review.

Standardised fronto-temporo-parietal flap technique accompanied by osteoclastic temporal decompression, and plastically enlargement of the dura-mater after durotomy was performed uni- or bilateral (35, 66). The median fronto-occipital diameter of craniotomy was at least 12 cm (57, 64). The indication for bilateral DC was the lack of ICP below values of 25 mmHg after the unilateral surgical procedure (67, 68). Bi-frontal craniectomy was not part of the general standard operating procedures of the neurosurgery departments and was therefore not used. The authors assume that the general guidelines and the in-house standard operation procedures have not changed over the years. The authors refrain from the repeated presentation of the extent of DC in this study compared to the previously presented study (57).

Although the chronological age does not correspond to any clinical homogeneity, we decided to divide the cohort into patients ≤65 years and > 65 years of age based on appropriate socio-economic (retirement age) and clinical applications (comorbidity) (69, 70). The OECD indicates that the normal retirement age in the OECD countries is approximately 64 years (69). Clinically, a higher likelihood of comorbidities and a higher risk of death can be associated with an age > 65 years (70). The group of older patients was subdivided again into younger old people (66 to ≤74 years) and elderly (≥75 years) (70).

### Data collection

The digitised and analogue patient archives were searched for patient-specific records. Patients were identified retrospectively using ICD10 codes (S00 to S09 and especially S06) and procedure codes (surgery and procedure codes, OPS, 5-01 and 5-02, especially 5-012, 5-020, 5-021). The surgery schedule of the relevant years and the image archives were searched twice to identify relevant surgeries. For further information, the documentation of the emergency services (paramedics and emergency physicians), the emergency department treatments, the primary and possible secondary in-hospital treatment as well as the reports of further treatment departments such as rehabilitation institutions and general practitioners’ reports were consulted. Patients’ own follow-up notes were used to assess outcome. If these were not available, telephone interviews were conducted with the most recent treating physicians, carers and relatives twice.

### Measurement of outcome

Outcome was described by the values of the Glasgow Outcome Scale (GOS) (55). The outcome was assessed at discharge and 12 months through clinical follow-up assessments or by telephone interviews. Unfortunately, there were drop-outs in follow-up: 3 (1.3%) patients for discharge outcome data (all in the group of patients ≤65 years of age = 2%) and 19 (8.5%) patients for 12 months outcome data (17 (11%) in the group of patients ≤65 years and 2 (3%) in >65 years of age). Sophisticated statistical measures, such as multiple imputation, were undertaken to replace the missing data and ensure a high-quality statistical analysis (71). In order to facilitate results interpretation, the five level scale was dichotomised in two different ways into the group of patients with unfavourable outcome and favourable outcome. The first method of dichotomisation was performed by sliding dichotomisation (Table 1) (72, 73). The second method is represented by simply dividing the patients with a GOS value of I to III for unfavourable outcome and GOS value of IV to V for favourable outcome (74). Both methods were compared in the aspects of their results. The results differed only marginally. However, due to recommendations of recent publications, we kept the sliding dichotomisation with prognostic bands for regression analysis (75). As the differences between the two methods were marginal and there are no recommendations for the use of sliding dichotomisation in ROC analyses in the literature, simple dichotomisation was used for the latter. Since the primary endpoint of this study was the outcome in older adults, who often have secondary diseases that require anticoagulants in their medication, laboratory values on admission were taken into account when calculating the outcome: Glucose in mg/dL, Partial Thrombin Time (PTT) in seconds, Platelet Count in giga/L, Fibrinogen in mg/dL, Leucocytes per μL, Haemoglobin (Hb) in g/dL, and Haematocrit in percent. Further, as cranioplasty may also influence the outcome of TBI and DC, the occurrence of cranioplasty was also taken into account in the statistical calculations (76).

**Table 1:** Sliding dichotomy based on three prognostic bands.

	Dead	VS	SD	MD	GR	Total
*GOS at discharge*						
Worst prognosis band	21 40%	**16** **30%**	**13** **24%**	**2** **4%**	**1** **2%**	53
Intermediate prognosis band	23 34%	18 27%	**24** **36%**	**2** **3%**	**0** **0%**	68
Best prognosis band	10 16%	15 24%	**22** **35%**	**15** **24%**	**1** **1%**	63
*GOS after 12 months*						
Worst prognosis band	32 58%	**3** **5%**	**11** **20%**	**2** **4%**	**7** **13%**	55
Intermediate prognosis band	24 44%	1 2%	**16** **29%**	**5** **9%**	**9** **16%**	55
Best prognosis band	13 22%	9 15%	7 12%	**8** **13%**	**23** **38%**	60

Prognostic bands were calculated using tertiles of the propensity score (PS) distribution; PS was calculated for both endpoints (GOS at discharge and after 12 months) using binary logistic regression for favourable outcome GOS IV & V based on baseline predictors age, motor score of the Glasgow Coma Scale (GCS), and Marshall score.

The categories for the GOS are Dead; Vegetative State (VS); Severe Disability (SD); Moderate Disability (MD); and Good Recovery (GR). The columns highlighted in bold correspond to the definition of favourable outcome according to the sliding dichotomisation approach. Bold values represent statistical significant results.

### Statistical analysis

Patients for whom no consciousness status, vital signs, procedural time markers or pre-operative imaging could be determined were excluded from the analysis (*N* = 2). The data were analysed with descriptive methods. The mean, standard deviation, median, and range were reported in the case of quantitative parameters and absolute and relative frequencies for the qualitative parameters. Knowing that GCS is an ordinally scaled value and should therefore be reported as median with range, we also provide the mean with standard deviation. This is because many international publications also report the GCS as a mean. This makes it easier to compare results. Explorative tests between interesting subsets were selected based on the underlying parameters. The presence of outliers was initially checked by descriptive methods. In case relevant outliers were present in a sense that the underlying data distribution is skewed, subsequent analyses followed a non-parametric approach when explorative hypothesis testing was done. The normal assumption with respect to the continuously scaled variables of the data set was checked on a graphical approach using histograms and quantile-comparison-plots. Given a sufficient sample size of the considered subsets, non-parametric tests such as Mann–Whitney-U and Kruskal-Wallis were performed. Group differences were also assessed by means of univariate and multivariable binary logistic regression analysis (95% confidence intervals included). Calculations for the receiver-operating-characteristics (ROC) curve as well as the area under the curve procedures were performed to identify cut-off values by means of maximising Youden’s index and minimising the closest top left value. Significance was established as *p* ≤ 0.05.

Age groups, time between trauma and surgery, disturbances of pupil function, GCS at admission, Marshall Score, mean arterial pressure (MAP) on admission, heart rate on admission, duration of stay at ICU, performed cranioplasty, as well as the duration of artificial ventilation on the occurrence of an unfavourable outcome were calculated. All statistical tests were analysed using the SPSS Statistics Software, Version 25 (IBM Corp. USA) and the R software (The R Foundation for Statistical Computing, Austria).

## Results

### Patient cohorts

In 18 years (2005 to 2022) of observation 223 patients were consecutively treated by DC after TBI. As a mean 12 (SD: 6.16) DC were performed each year. The majority of patients were male, *N* = 163 (73.1%). Mean age during the observation period was 49.9 (SD: 21.38) years, range 1 to 93 years. There was no significant difference in the mean age over the years (ANOVA and Kruskal-Wallis-H test). There were 157 (70.4%) patients in an age of ≤65 years and 66 (29.6%) patients in an age of >65 years. The proportion of patients >65 years of age (*N* = 66) was divided into the age range 66 to ≤74 years with *N* = 33 (14.8%) and ≥ 75 years with *N* = 33 (14.8%). Bilateral DC was performed in 10 (4.5%) patients.

### Clinical characteristics

Clinical characteristics are outlined in Table 2. The mean time between trauma and surgery was 28.8 (SD: 81.48) hours. One patient was operated after 936 h (39 days). Without this, the mean time was 23.8 (SD: 45.02) hours. There was a significant difference in the time between trauma and surgical treatment between the age groups (*p* = 0.007 for ≤65 years versus >65 years and *p* = 0.026 for the subgroup analysis of >65 years, Mann–Whitney-U test). However, younger (≤65 years) patients were treated earlier than older (>65 years) patients (Table 2).

**Table 2:** Clinical characteristics (*N* = 223).

	Total (%)	Age ≤ 65 years	Age > 65 years	*p*	Age 66–≤74 years	Age ≥ 75 years	*p*
*N* (%)	223 (100)	157 (70.4)	66 (29.6)	<0.001	33 (14.8)	33 (14.8)	>0.05
Gender							
Female	60 (26.9)	34 (21.7)	26 (39.4)	0.008	12 (36.4)	14 (42.4)	0.401
Male	163 (73.1)	123 (78.3)	40 (60.6)		21 (63.6)	19 (57.6)	
*Age (years)*							
Mean (SD)	49.9 (21.38)	39.6 (16.70)	74.2 (5.94)	<0.001	69.4 (2.74)	79.1 (3.95)	0.158
Median (Range)	52.0 (1–93)	43.0 (1–65)	74.5 (66–93)		69.0 (66–74)	78 (75–93)	
Rate of in-hospital death, *N* (%)	65 (29.1)	37 (23.6)	28 (42.4)	0.006	11 (33.3)	17 (51.5)	0.213
*Time between trauma to surgery (h, SD)^a^*							
Mean (SD)	28.8 (81.48)	27.8 (90.43)	31.0 (58.30)	0.007	42.7 (64.54)	21.2 (51.53)	0.026
Median (Range)	7 (1–936)	5 (1–936)	11 (1–288)		13 (1–216)	6 (1–288)	
Mean (SD) without case (936 h)	23.8 (45.02)	20.4 (37.04)	31.0 (58.30)	0.005			
Median (Range)	7 (1–288)	5 (1–216)	11 (1–288)				
Rate of pupil function disturbance, *N* (%)	94 (42.2)	67 (42.7)	27 (40.9)	0.433	13 (39.4)	14 (42.4)	0.543
*Glasgow Coma Scale at admission*							
Total				<0.001			0.774
Mean (SD)	5.0 (3.89)	4.4 (3.28)	6.6 (4.68)		6.6 (4.96)	6.7 (4.46)	
Median (Range)	3 (3–15)	3 (3–15)	3 (3–15)		3 (3–15)	4 (3–15)	
Eye Opening				<0.001			0.664
Mean (SD)	1.5 (1.04)	1.3 (0.83)	2.0 (1.31)		1.9 (1.32)	2.0 (1.32)	
Median (Range)	1 (1–5)	1 (1–5)	1 (1–4)		1 (1–4)	1 (1–4)	
Motor response				<0.001			0.611
Mean (SD)	1.9 (1.81)	1.6 (1.53)	2.7 (2.14)		2.6 (2.24)	2.8 (2.09)	
Median (Range)	1 (1–6)	1 (1–6)	1 (1–6)		1 (1–6)	2 (1–6)	
Verbal Response				<0.001			0.974
Mean (SD)	1.5 (1.18)	1.3 (0.96)	2.0 (1.50)		2.0 (1.59)	1.9 (1.42)	
Median (Range)	1 (1–6)	1 (1–5)	1 (1–6)		1 (1–5)	2 (1–6)	
Marshall score							
Mean (SD)	4.3 (1.26)	4.2 (1.24)	4.5 (1.28)	0.208	4.5 (1.20)	4.5 (1.37)	0.838
Median (Range)	4 (2–6)	4 (2–6)	4 (2–6)		4 (2–6)	4 (2–6)	
*Vital signs at admission (Mean, SD)*							
Initial MAP (mmHg)	89.9 (35.08)	85.0 (37.07)	102.0 (26.24)	<0.001	104.4 (17.36)	99.5 (33.42)	0.674
Initial Pulse (beats/min)	85.9 (24.76)	86.5 (25.91)	84,4 (21.90)	0.523	81.8 (23.95)	87.2 (19.59)	0.129
Length of ICU in days (Mean, SD)	12.7 (11.40)	14.3(13.48)	8.9 (8.09)	<0.001	11.1 (9.10)	6.7 (6.31)	0.05
*Additional injuries*							
Thorax	25 (11.2)	21 (13,4)	4 (6.1)	0.085	0 (0)	4 (12.1)	0.057
Abdomen	6 (2.7)	6 (3.8)	0 (0)	0.118	0 (0)	0 (0)	
Extremities	18 (8.1)	15 (9.6)	3 (4.5)	0.163	0 (0)	3 (9.1)	0.119
Spine	17 (7.6)	12 (7.6)	5 (7.6)	0.613	2 (6.1)	3 (9.1)	0.500

a One case had surgery 936 h after admission due to secondary brain edema (ICU=Intensive Care Unit).

The mean GCS score on admission was 5.0 (SD: 3.89) and the median was 3 (3–15). There is a significant difference (*p* < 0.001, Mann–Whitney-U test) between the cohorts stratified according to age ≤ 65 years [GCS = 4.4 (SD: 3.28)] and > 65 years [GCS = 6.6 (SD: 4.68)]. Older patients showed a higher mean GCS value. In the group of patients >65 years of age, there was no significant difference in GCS values between patients 66 to ≤74 years and patients ≥75 years (Table 2).

Ninety-four (42.2%) patients showed a disorder of the pupillomotor function on admission. The initial computed tomography (CT) showed a mean degree of injury of 4.3 (SD: 1.26) according to the Marshall Score. There were no significant difference between the age groups.

The initial mean MAP was 89.9 (SD: 35.08) mmHg. Patients aged ≤65 years had a lower mean MAP (85.0; SD: 37.07) than patients aged >65 years (102.0; SD: 26.24) (*p* < 0.001, Mann–Whitney-U test). There were no significant differences in MAP within the older patients. The initial mean heart rate on admission was 85.9 (SD: 24.76) beats per minute and showed no significant difference between the age groups.

The mean length of stay at the intensive care unit (ICU) was 12.7 (SD: 11.40) days. After DC, patients aged ≤65 years remained at ICU for a mean of 14.3 (SD: 13.48) days and patients >65 years had a mean of 8.9 (SD: 8.09) days (*p* < 0.001, Mann–Whitney-U test). This difference becomes clearer within the group of older patients. Patients aged 66 to ≤74 years stayed with a mean of 11.1 (SD: 9.10) days and patients ≥75 years stayed 6.7 (SD: 6.31) days at the ICU (*p* = 0.050, Mann–Whitney-U test). Two hundred and seven (92.8%) patients had to be ventilated postoperatively or perioperatively. The mean duration of artificial ventilation was 170.7 (SD: 179.66) hours. Patients aged ≤65 years (*N* = 144, 69.6%) were ventilated for 196.2 (SD: 190.11) hours in mean and patients >65 years (*N* = 63, 30.4%) were ventilated for 112.4 (SD: 137.58) hours (*p* = 0.001, Mann–Whitney-U test) in mean. Patients 66 to ≤74 years of age (*N* = 31, 49.2%) had a mean of 152.4 (SD: 174.01) hours and patients ≥75 years (*N* = 32, 50.8%) had a mean of 73.7 (SD: 73.49) hours on ventilation (*p* = 0.211, Mann–Whitney-U test).

Cranioplasty was performed in 104 (46.6%) patients. Ninety (57.3%) younger patients (≤65 years) and 14 (21.2%) older patients (>65 years) received a cranioplasty (*p* < 0.001, Fisher Exact Test and Mann–Whitney-U test). There were also significant differences in the differentiation of the group of older patients. Eleven (33.3%) patients aged 66 to ≤74 years and three (9.1%) of the older patients (≥75 years) received a cranioplasty (*p* = 0.017, Mann–Whitney-U test). Patients who underwent cranioplasty had a GOS after 12 months: GOS I (4%), GOS II (13%), GOS III (25%), GOS IV (19%) and GOS V (39%) (*p* = 0.001, Kruskal-Wallis test).

### Laboratory tests

**Figure 1:**
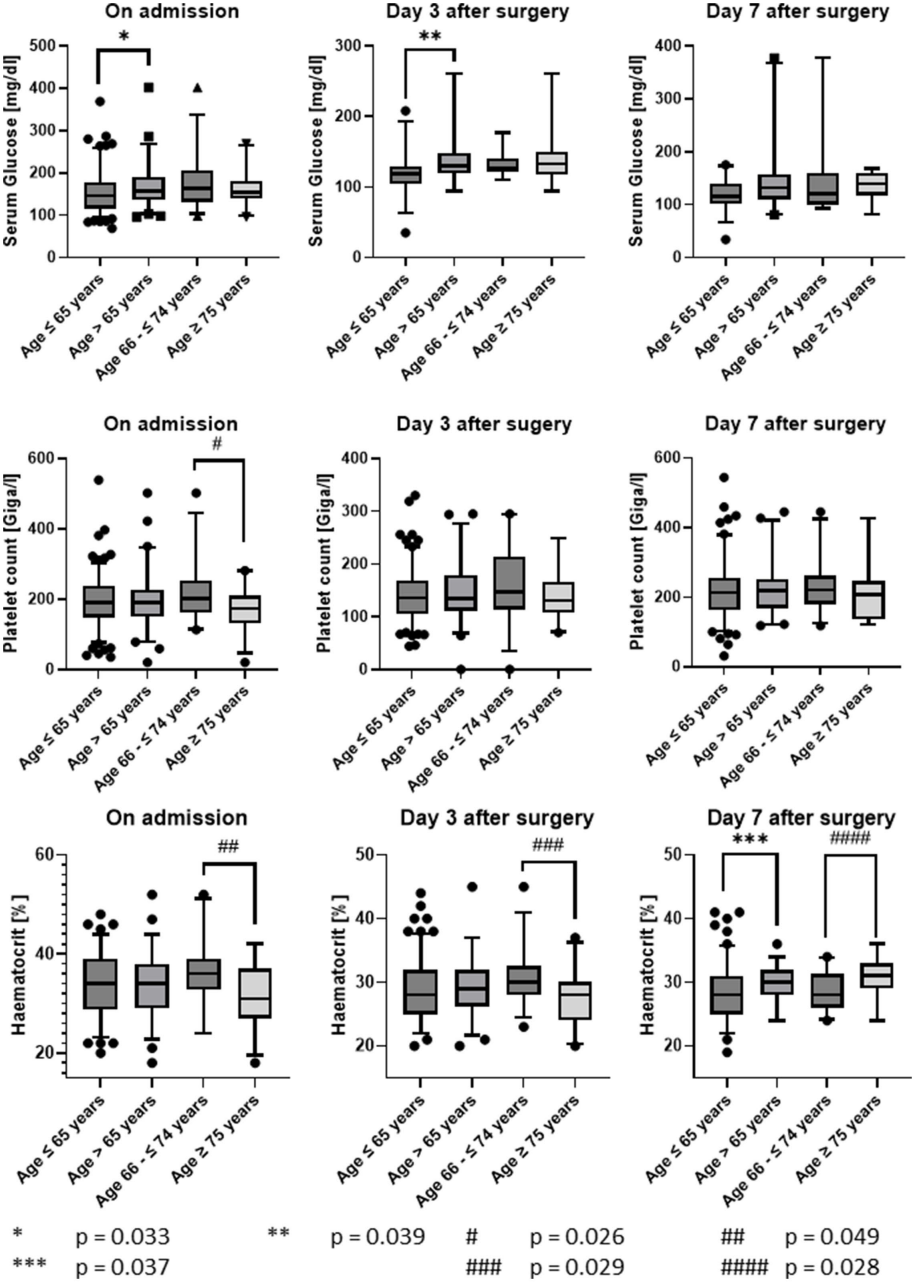
Course of laboratory tests.

The blood laboratory tests showed significant differences between the age groups in the values for glucose (*p* = 0.33 on admission and 0.039 at day 3 after surgery, Mann–Whitney-U test), platelet count (*p* = 0.026 on admission, Mann–Whitney-U test), and haematocrit (*p* = 0.049 on admission, *p* = 0.029 at day 3, *p* = 0.028 at day 7 after surgery, Mann–Whitney-U test). Patients aged ≤65 years had lower blood glucose levels on admission and day 3 after surgery than patients >65 years. Patients aged 66 to ≤74 years had higher platelet counts than patients aged ≥75 years on admission. With regard to haematocrit, patients aged ≤65 years showed significantly lower values than patients aged >65 years at day 7 after surgery. In the group of older patients, patients 66 to ≤74 years had lower values on the day of admission, higher values on the third day after surgery and significantly lower values again at day 7 after surgery compared to patients aged ≥75 years (Figure 1). Clinically relevant statistical calculations for the partial thromboplastin time (PTT), Quick value, fibrinogen, leukocyte count or haemoglobin content remain without significant differences.

### Outcome

**Figure 2:**
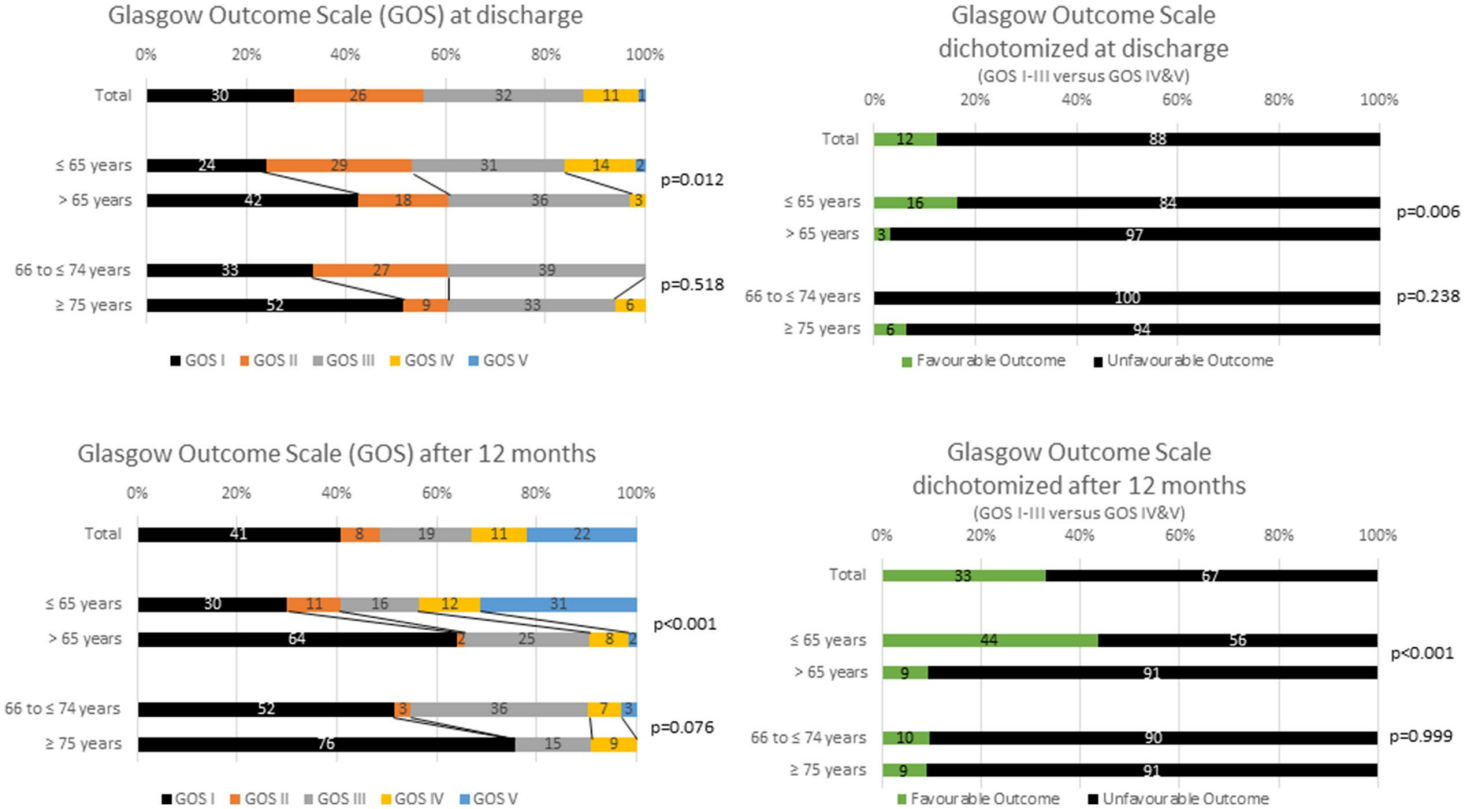
Outcome according to the Glasgow Outcome Scale (Favourable Outcome = Grade IV and V, Unfavourable Outcome = Grade I to III).

More than one-fourth of patients died during the primary inpatient stay (*N* = 65, 29.5%). There is a significant difference between the age groups (Figure 2). Of 157 patients aged ≤65 years, 37 (23.6%) died and of 66 patients aged >65 years, 28 (42.4%) died during the primary clinical stay (*p* = 0.006, Chi-square test). In the group of the older patients (*N* = 66), 11 (33.3%) patients died at the age of 66 to ≤74 years and 17 (51.5%) at an age of ≥75 years (*p* = 0.213, Chi-square test) (Table 2).

At discharge, 1.4% had a very good and 10.9% a good outcome (GOS). Approximately 88% had an unfavourable outcome (GOS I to III). The stratification into patients ≤65 years and > 65 years showed a significant difference in favour for the younger patients (*p* = 0.0012, Kruskal-Wallis test). A further breakdown of the elderly does not reveal any significant difference either (Figure 2).

Further, 40.7% of the patients died after 12 months. Approximately one-third (32.9%) had a good or very good outcome according to GOS. In the age group >65 years (64.1%) mortality was significantly higher than in the age group ≤65 years (30%) (*p* < 0.001, Kruskal-Wallis test and Chi-square test). A very good treatment result occurred more frequently in the group of ≤65 years old (31.4%) than in the group of >65 years old (1.6%) (*p* < 0.001, Kruskal-Wallis test and Chi-square test). Fatal outcome was not significantly less frequent in patients aged 66 to ≤ years (51.6%) compared to patients aged ≥75 years of age (75.8%) (Figure 2).

In a statistical model of univariate binary logistic regression analysis, the factors influencing significantly the favourable outcome (sliding dichotomization) after 12 months are represented by the variables age (OR 0.937, *p* = 0.007, CI 95%: 0.894–0.981 in worst prognostic band), Cranioplasty in worst, intermediate best prognostic band (Table 3). In a multiple regression analysis approach, the factor of cranioplasty was confirmed (Table 3).

**Table 3:** Results of univariate and multivariable binary logistic regression analysis for favourable outcome^a^ at discharge and after 12 months within prognostic bands^b^.

Model	Univariate			Multivariable		
	OR	95% CI	*p*-value	OR	95% CI	*p*-value
**Favourable outcome (GOS) at discharge**						
*Worst prognostic band*						
Age	0.976	0.934–1.020	0.273	1.019	0.963–1.080	0.494
Trauma-surgery time	1.006	0.985–1.028	0.564	0.998	0.965–1.032	0.896
Duration artificial ventilation	1.005	1.000–1.011	**0.039**	1.006	0.999–1.012	0.074
Cranioplastic (yes vs. no)	15.474	1.751–136.780	**0.015**	17.666	1.566–199.311	**0.021**
*Intermediate prognostic band*						
Age	1.016	0.988–1.044	0.267	1.028	0.991–1.067	0.137
Trauma-surgery time	1.012	0.996–1.028	0.151	1.010	0.992–1.027	0.272
Duration artificial ventilation	0.999	0.996–1.002	0.355	0.996	0.992–1.001	0.088
Cranioplastic (yes vs. no)	4.722	1.594–13.994	**0.006**	12.822	2.844–57.818	**0.001**
*Best prognostic band*						
Age	0.999	0.972–1.028	0.967	1.012	0.978–1.047	0.137
Trauma-surgery time	0.996	0.983–1.008	0.467	0.994	0.980–1.008	0.272
Duration artificial ventilation	0.999	0.995–1.002	0.457	1.000	0.996–1.004	0.088
Cranioplastic (yes vs. no)	7.768	2.388–25.266	**0.001**	8.873	2.501–31.480	**0.001**
**Favourable outcome (GOS) after 12 months**						
*Worst prognostic band*						
Age	0.937	0.894–0.981	**0.007**	0.960	0.910–1.013	0.135
Trauma-surgery time	1.005	0.983–1.027	0.629	1.004	0.974–1.035	0.777
Heart rate	0.987	0.963–1.011	0.282	0.989	0.963–1.015	0.388
Cranioplastic (yes vs. no)	8.501	1.941–37.234	**0.005**	5.508	1.030–29.470	**0.046**
*Intermediate prognostic band*						
Age	0.987	0.955–1.020	0.438	0.993	0.943–1.046	0.794
Trauma-surgery time	1.001	0.989–1.014	0.871	1.007	0.991–1.022	0.382
Heart rate	0.976	0.963–1.010	0.258	0.997	0.955–1.040	0.877
Cranioplastic (yes vs. no)	32.946	6.012–180.539	**0.001**	36.960	5.851–233.463	**0.001**
*Best prognostic band*						
Age	1.018	0.983–1.055	0.313	1.009	0.963–1.056	0.794
Trauma-surgery time	1.013	0.996–1.031	0.139	1.017	0.995–1.040	0.382
Heart rate	0.976	0.951–1.002	0.067	0.961	0.925–0.999	0.877
Cranioplastic (yes vs. no)	8.747	2.536–30.176	**0.001**	10.228	2.336–44.780	**0.001**

a Glasgow Outcome Scale (GOS) was used as outcome measure, whereas favourable or unfavourable outcome was defined according to a sliding dichotomy approach [(I) Favourable outcome at discharge: worst prognostic band: GOS II-V, intermediate prognostic band: GOS III-V, best prognostic band: GOS III-V; (II) Favourable outcome after 12 months: worst prognostic band: GOS II-V, intermediate prognostic band: GOS III-V, best prognostic band: GOS IV & V].

b Prognostic bands were calculated using tertiles of the propensity score (PS) distribution; PS was calculated for both endpoints (GOS at discharge and after 12 months) using binary logistic regression for favourable outcome GOS IV & V based on baseline predictors age, motor score of the Glasgow Coma Scale (GCS), and Marshall score.

OR = odds ratio; 95% CI = 95% confidence interval; age in years; time between trauma and surgery in hours; duration of artificial ventilation in hours; heart rate in beats per minute.

### Thresholds for outcome

**Figure 3:**
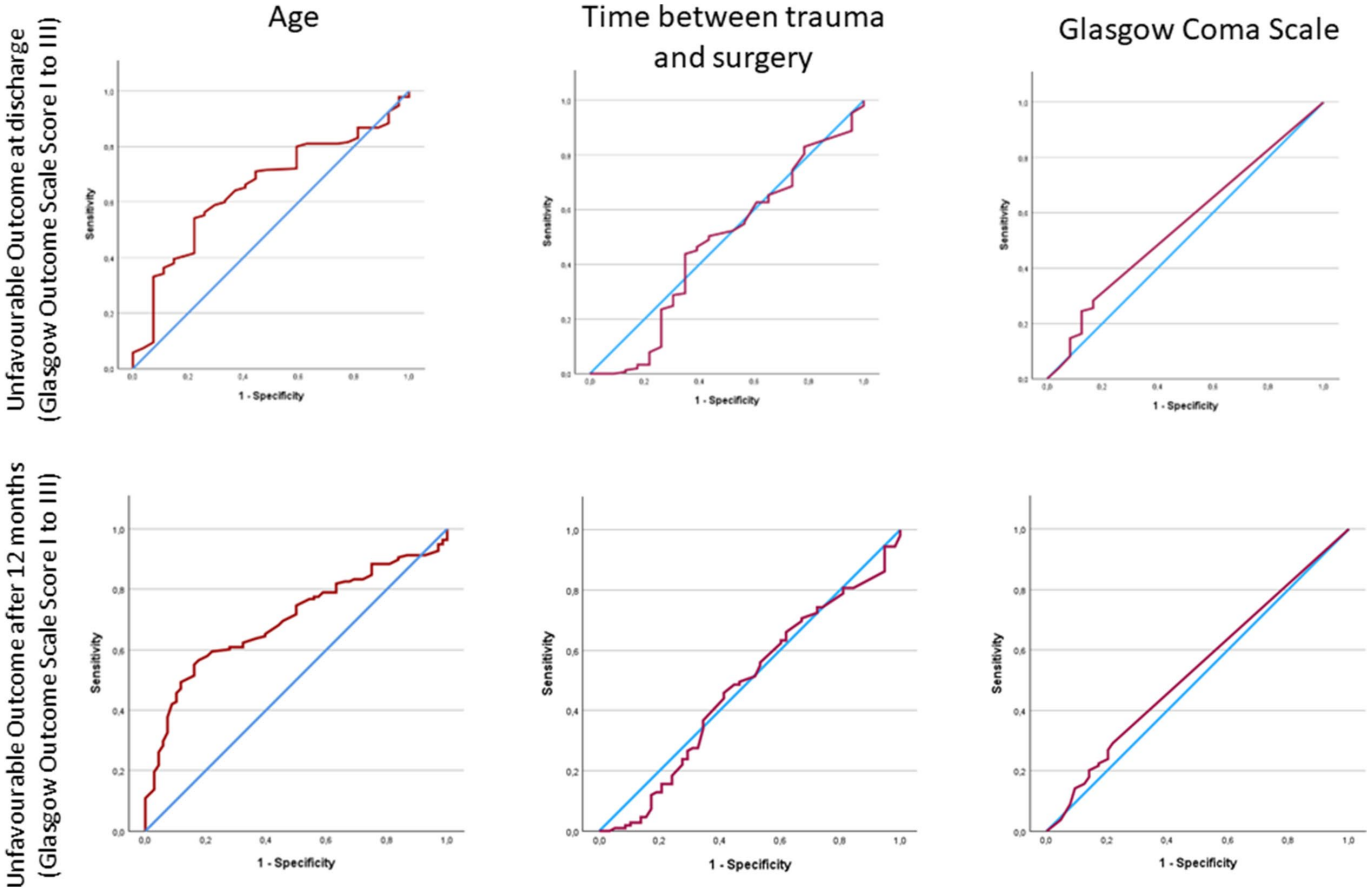
Area under the curve (AUC) calculations for discharge and 12 months outcome.

Due to the sparse statistical yield factors influencing the outcome at discharge and after 12 months, a receiver operating characteristic analysis (ROC) was performed by using the simple dichotomisation (unfavourable outcome = GOS I to III). For patients after severe TBI and DC an age of 55.5 years, time between trauma and surgery of 8.25 h and a GCS of 4 for the outcome after 12 months and an age of 51.5 years, time between trauma and surgery of 9.5 h and a GCS of 6 (Youden’s Index) or 4 (Closest top left value) for the outcome at discharge could be identified as a threshold for unfavourable outcome. Nevertheless, the variable “age” had highest individual AUC calculations compared to the other: AUC (95% CI) = 0.7 (Figure 3; Table 4).

**Table 4:** Diagnostic accuracy of predictor variables: age, time between symptom onset or trauma and surgery, and level of consciousness by Glasgow Coma Scale (GCS) (*N* = 223, estimation of optimal cut-off by means of maximisation of Youden’s index).

	Age (years)	Time between symptom start or trauma and surgery (hours)	GCS
*ROC analysis for TBI patients, unfavourable outcome after 12 months*			
AUC (95% CI)	0.70 (0.63, 0.77)	0.48 (0.39, 0.57)	0.53 (0.45, 0.62)
Cut-off Youden’s / Closest top left	55.5	8.25	4
Sensitivity	0.57	0.46	0.29
Specificity	0.18	0.41	0.22
*p*	<0.001	0.671	0.429
*ROC analysis for TBI patients, unfavourable outcome at discharge*			
AUC (95% CI)	0.65 (0.55, 0.75)	0.47 (0.34, 0.61)	0.56 (0.44, 0.67)
Cut-off	51.5	9.5	6 / *4*
Sensitivity	0.54	0.44	0.25 / *0.28*
Specificity	0.22	0.35	0.13 / 0.17
*p*	0.003	0.708	0.364

Bold values represent statistical significant results.

## Discussion

The aim of the study was to describe differences in clinical outcome at discharge and at 12 months after severe TBI and DC in younger and older patients. There was a significant difference in the outcome in younger (≤ 65 years) and older (> 65 years) patients at discharge and after 12 months. In our analysis, patients who are >65 years old had an overall risk of a poor outcome after TBI and DC and 41% of the patients died after 12 months. Only 9% of those >65 years old have a favourable outcome, while one-quarter are dependent on help in everyday activities. For the unfavourable outcome, cut-off values for age, time between trauma and surgery and GCS could be calculated. However, the GCS is not suitable as a decision parameter, especially for the elderly, due to its low sensitivity to outcome (77), its ability to be influenced by sedation in a prehospital environment, cognitive and language deficits, and impaired consciousness from alcohol and drugs. Further, the time between symptom start or trauma and surgery is not always influenced by medical measures. We therefore discuss the chronological age in the following.

### Outcome after TBI in older adults

In patients >65 years of age the pooled annual incidence for TBI is 166 per 100.00 (CI 95%: 143–194) (38). There are indications that the mean age of TBI patients has increased steadily over the past 50 years (13). One reason for this is seen in the fact that with increasing life expectancy there is increasing mobility in older people (41). Falls are the main reason for TBI in the elderly (13, 31). Using the meta-analysis published by Stein et al., they show that medical progress has not consistently led to an improvement in mortality after TBI. Mortality stagnated between 1930 and 1970. Between 1970 and 1990, the introduction of CT and improvements in intensive care medicine led to a reduction in mortality after TBI. After 1990 there was a stagnation of the mortality rates after TBI. One possible explanation could be the increase in older patients with a higher likelihood of mortality after TBI with slowly improving medical conditions (78). This shows the possible influence and the importance of an aging society for the increasing proportion of older people in in-hospital treatment for TBI.

Especially in older adults (age > 65 years) the outcome is related to the severity of the TBI (39, 79, 80). When describing the severity of the TBI, it makes sense to proceed uniformly (81). Because especially in older adults, a higher GCS is more often found after TBI than in the younger patients (13, 31). This fact is also reflected in our data. Nevertheless, better GCS results often in a higher mortality rate (22, 31, 82). Maiden et al. reported a mortality rate of 79% in their 10-year study (83). McIntyre et al. described the outcome as 7.9% for favourable (CI: 5.3–11.8), 13.8% for unfavourable (CI: 10–18.8%) and 79.3% for fatal (CI: 73.2–84.4%) (84). The comparison of these results, which apply to all patients >65 years of age and without therapeutic subgroup analysis, with those of our study after TBI and DC show a difference. In our study and in view of our socio-cultural background with a health-care system in a high-income country, fewer older patients died and more patients achieved a favourable outcome. Our results are more in line with other observations after TBI and DC (51). Ramanathan et al. report about a fatality rate of 2,386 (58%) in 3895 elderly individuals after TBI (13). In their study fatality rate decreased from 65% in 1992 to 53% in 2009. Hazare et al. report about an in-hospital rate of 22% and a 6-months rate of mortality of 56% (85). Eom et al. establish a relationship between the mortality rate and the trauma mechanism in such a way that they postulate a mortality rate of 4% for older adults after a fall and 12% after a road traffic accident (79). The authors think it is very sensible to scientifically investigate the further developments in in-hospital outcome of older patients after TBI, since the social development of demographic change seems to continue.

Pre-existing illnesses and comorbidities should play a significant role in this context, because, for example, coagulation disorders can significantly influence the outcome (86, 87). Hecht et al. were able to show that the presence of anticoagulant therapy prior to TBI significantly increases the risk of mortality (88). We were able to show that there are significant differences in coagulation values between younger and older patients. These differences in coagulation must be taken into account to enable the best possible outcomes (87, 89).

In our study, cranioplasty was identified as an important factor influencing the outcome. Here, our study confirms the results of others (90–92). However, significant differences are known in how the outcome after TBI and cranioplasty is described and how the cohort composition takes place to describe the outcome (93, 94). It is not uncommon for selection errors to occur, as cranioplasty particularly supports those who have a very good potential for a favourable outcome regardless of the cranioplasty to achieve a better outcome (93). This was also observed in our collective, as significantly more cranioplasties were performed in patients with a higher GOS value than in patients with a lower GOS value. It remains difficult to discuss whether the performance of a cranioplasty can be regarded as an independent factor for the outcome, or whether there is a connection between a subjectively and objectively expected (favourable) outcome and the performance of a cranioplasty (94).

### Decompressive craniectomy in older adults

In our study on the use of DC after severe TBI, approximately one third of all patients were > 65 years old. Therapy of the refractory ICP represents a common final pathway of many cerebral pathological conditions (57). DC is effective in decreasing refractory ICP and in reducing of space occupying intracranial effects such as brainstem compression, basal cistern compression, and midline shift, in part in combination with evacuation of space occupants such as, e.g., haemorrhages (49, 57, 95). The DC is superior over a non-surgical approach in terms of survival after severe TBI (49, 96). The use of DC in older adults can therefore be considered. However, the evidence of a benefit of DC for the patients’ outcome beside survival is under debate. Several studies have been performed to get a better understanding herein (97–102). Up to now, there is only stressable evidence for a better outcome in patients after ischemic stroke (103). Studies in patients after TBI, subarachnoid haemorrhage, intracerebral haemorrhage or other like inflammatory brain diseases failed to give straight clinical advises or gave more further questions to debate on (96, 100, 104).

Knowledge about the outcome of older adults after severe TBI and DC is limited (51, 105–107). Kim et al. report in their propensity score matching analysis that DC was able to reduce the mortality rate in the treatment group after 6 months (62% versus 52%, *p* = 0.179). However, favourable outcome was less common in the DC group compared with the non-DC-group (12% versus 18%, *p* = 0.296). For this study, however, the question must be asked as to how the decision was made not to perform DC. This may be a selection error that reflects the difficulty of deciding in favour of or against surgery. Paldor et al. report that less than 2% out of their collective of older adults treated with DC had a good outcome 6 months after discharge (53). There are no guidelines for the treatment of severe TBI and DC in older adults. The decision to treat aggressively or conservatively or to withhold treatment in older adults is a difficult balance between medical knowledge, experience and assessment of the physicians on the (108) one hand and the (presumed) will of the patient on the other (109). Nevertheless, Skaansar et al. showed that the intensity of treatment in patients with TBI decreases with increasing age, and that low intensity of management is associated with an increased risk of 30-day mortality (110).

Little is known about the timing of DC in older adults with a different intracranial physiology than in younger patients (105, 111–113). However, there is evidence in the literature that the timing of surgical treatment is related to the severity of the injury rather than being a predictor of outcome (114). However, these studies rarely include older adults who have had a TBI and for whom physicians and/or relatives have decided against surgery. With an increasing proportion of older adults in the society and an increasing proportion of older adults being treated in-hospital for TBI, it is interesting whether the proportion of patients in whom DC is to be considered will also increase. This aspects should also be scientifically observed in connection with the outcome. DC is understood as a rescue therapy or final-tier treatment (66). However, in the context of timing of surgery and the time between trauma and surgery, the assessment of when the moment of final-trier has come can be considered differently (35, 96, 104, 115–118). One approach might be to perform the DC as early as possible. This in turn means that a very early-onset DC at an early time point of pathophysiology could ensure survival and good outcome after TBI. In this approach, it must be admitted that patients who are treated with DC, who would not have reached the pathophysiological end-point of refractory elevated ICP even without surgery, were thus over-treated (119).

### Decision making for surgery in older adults

In our study, the parameters age, time after trauma and the GCS value in the ROC analysis were analysed with regard to a distinguishing feature for the outcome. The chronological age showed the better values in terms of Area Under the Curve (AUC), sensitivity and specificity. A growing population of older adults in a society also means that more surgical procedures are being performed on older adults (120). As a consequence of improvements in surgery, anaesthesia and intensive care medicine, as well as improved clinical risk management and the increased demand of older adults to be able to experience complex medical interventions, decision-making for a surgery has developed into a complex and multidimensional process (121). Older adults with a positive discrepancy between biological and chronological age in particular decide to undergo surgery due to a life-threatening illness. They often see their decision as having no alternative, but give little thought to how they might live in the future, e.g., in the event of complications or unfavourable outcome (122). For older adults who are no longer able to participate in the decision-making process for or against surgery due to an acute illness or injury, the presumed will is very important (122). This can be expressed by means of an advance directive from the patient themselves or via relatives or close persons. However, the wishes of close family members also play a role in the decision-making process and can influence the expression of presumed will in such a way that the person’s own wishes are expressed instead of those of the patient. This expression of presumed will is influenced by age, medical and ethical knowledge as well as the working and social environment (123). However, such human and understandable behaviour needs to be weighed against the scientific evidence on the outcomes of older adults after TBI when making decisions.

Further, increased age is attributed to an increased postoperative risk of complications and a poorer outcome compared to younger patients (40, 84, 124–126). Physical and cognitive recovery is possible and uncertain in older adults (127). Current studies, however, distinguish between chronological age and frailty, which is described as an age-related cumulative decline in the functionality of physiological systems (128). Frailty is considered as a better predictor of morbidity and mortality after surgery (129, 130). The main difference between the concept of age and the concept of frailty in terms of mortality and morbidity after surgery is that not all patients of a chronological age have the same risk (120). However, there is currently no short, standardised, multidimensional assessment for determining frailty and especially not for emergency situations (128, 131). As already described, there are numerous studies on the relationship between age and outcome after TBI, with increased age usually being associated with a poorer outcome. In contrast, studies on the influence of frailty on the outcome after TBI are currently still very limited (132, 133). Nevertheless, it can be seen that higher frailty degrees are associated with an increased risk of in-hospital death, complications, prolonged hospital stay and unfavourable outcome (132, 134). Tang et al. reported that in their study frailty was even an outcome predictor independent of age and severity of TBI (135). Due to the increasing social importance of older adults and the growing importance of the difference between chronological and biological age, as well as the proven partially independent influence of frailty on the outcome of older adults after TBI, the authors suggest that the pathological condition of TBI in older adults should be addressed to agree on an appropriate frailty index. In our collective, we were not able to identify any further differences in outcome in the subgroup analysis of older adults. This also confirms other results (13). Unfortunately, due to the retrospective nature of our study, it is difficult to assign an appropriate frailty index. We agree with other authors in the field, frailty should be taken into account in future studies of TBI in older adults to reflect the heterogeneity of this patient group in terms of opportunities and risks (chronological versus biological age) and the decision making for or against TBI surgery in older adults (136).

### Limitations

The study presented has the main limitation of retrospective nature. This is particularly true for prehospital care with important data that could provide further insight into the patient’s initial condition and initial care. Further, the coagulation values cannot be adequately discussed due to a lack of detailed information on blood product administration. However, they are not the main subject of this study and require a separate prospective study. There were drop-outs in follow-up. This problem is associated with the distinct group size, different statistical utility and power, the susceptibility to clinical selection errors, and follow-up reporting errors. Nevertheless, the results of this study benefit from a non-small group size and from complex statistical and mathematical reasoning. However, elaborate statistical calculations were made. The resulting AUC values and the respective combination of sensitivity and specificity do not represent a high value for a prognosis (AUC < 0.75). Cut-off values can be calculated but given the heterogeneity of the patients (comorbidities and injury patterns), they do not have an excellent ability to discriminate. Therefore, the distinction between patients ≤65 years and > 65 years according to the outcome appears to be more meaningful. Finally, the study refers to an everyday occurrence with a minimum of available information in terms of age, level of consciousness based in the GCS and the time between trauma and DC. For the application of the DC after TBI, we were able to give statistical aids in a retrospective approach with regard to a cautiously judged threshold for age and time after trauma. We were also able to show that the outcome of older patients after TBI and DC is predominantly poor. The individual decision-making for older patients should, however, be made in the context of the overall injury including additional and previous illnesses as well as the (presumed) patient’s will. Even if the TBI is survived, there is a high probability that there is a dependency on third parties for everyday activities.

## Conclusion

Not all patients >65 years of age die from TBI and after DC. However, the likelihood of being in need of care is high. It is to be expected that as demographic change progresses, more older patients with TBI will have to be treated. Thus, when considering resource allocation for the application of DC in those, patients’ will, proxies capacities, the socio-economic and health-care system background of a society must always be considered. The further changes of societies should be scientifically observed prospectively.

## Data availability statement

The original contributions presented in the study are included in the article/supplementary material, further inquiries can be directed to the corresponding author.

## Ethics statement

The studies involving humans were approved by Ethics Committee of the University of Ulm (No. 439/17 and No. 63/23). The studies were conducted in accordance with the local legislation and institutional requirements. The ethics committee/institutional review board waived the requirement of written informed consent for participation from the participants or the participants’ legal guardians/next of kin because the study was designed and conducted as a retrospective investigation.

## Author contributions

TK: Conceptualization, Formal analysis, Investigation, Methodology, Resources, Software, Supervision, Validation, Visualization, Writing – original draft, Writing – review & editing. SJ: Formal analysis, Investigation, Methodology, Validation, Writing – review & editing. FraS: Formal analysis, Investigation, Validation, Writing – review & editing. FreS: Formal analysis, Investigation, Validation, Writing – review & editing. DW: Formal analysis, Validation, Writing – original draft, Writing – review & editing. SG: Formal analysis, Investigation, Validation, Writing – review & editing. EB: Formal analysis, Validation, Writing – original draft, Writing – review & editing. BM: Conceptualization, Formal analysis, Methodology, Validation, Writing – original draft, Writing – review & editing. MO: Formal analysis, Investigation, Validation, Writing – original draft, Writing – review & editing. AP: Conceptualization, Methodology, Supervision, Validation, Writing – original draft, Writing – review & editing.
